# International visualization analysis of research hotspots and development trends in the study of clinical decision support systems utilizing CiteSpace

**DOI:** 10.3389/fmed.2025.1546611

**Published:** 2025-04-28

**Authors:** Meixuan Song, Dong Liu

**Affiliations:** ^1^Department of General Surgery (Gastrointestinal Surgery), The Affiliated Hospital of Southwest Medical University, Luzhou, China; ^2^Department of Pediatrics, The Affiliated Hospital of Southwest Medical University, Luzhou, China; ^3^Sichuan Clinical Research Center for Birth Defects, Luzhou, China

**Keywords:** clinical decision support system, hotspots, development trends, visual analysis, CiteSpace

## Abstract

**Objective:**

This study aims to elucidate the current status and trends in clinical decision support systems (CDSS). It will analyze the direction of research development in this field and provide valuable references for future research and the application of CDSS.

**Methods:**

We conducted a search of the Web of Science Core Collection database from January 2014 to May 2024 to identify relevant literature on clinical decision support systems. CiteSpace (6.2. R4) software was utilized to visualize and analyze various aspects of the included literature, including publication volume, country of origin, authors, institutions, cited literature, keywords, and keyword clustering, and to generate corresponding graphs.

**Results:**

A total of 2,668 articles were ultimately included in this study. The scholar with the highest number of publications is Professor Adam from the Department of Biomedical Information at Vanderbilt University in the United States. The top five countries contributing to this research are the United States, the United Kingdom, Germany, the Netherlands, and China. Based on an analysis of cited literature and keyword clustering, the research primarily focuses on predicting biochemical recurrence, cardiovascular disease, clinical guidelines, evidence-based computerized decision support systems, and intensive care units. The prominent topics in this field include artificial intelligence, natural language processing, venous thromboembolism, user-centered design, and emergency medicine.

**Conclusion:**

Research on CDSS is demonstrating an upward trend and shows promising development prospects. Artificial intelligence, natural language processing, and user-centered design are the future trends.

## 1 Introduction

Clinical Decision Support Systems (CDSS) are computer-aided information systems that assist medical personnel in clinical diagnosis and treatment activities through information technology. By integrating medical knowledge with patient information, CDSS supports clinical decision-making (1). These systems offer intelligent services, including disease screening and diagnosis, disease warnings, medical order monitoring, adverse drug event alerts, and nursing support in healthcare (2–5). CDSS has been successfully implemented in various fields, such as venous thromboembolism, cardiovascular disease, and cancer, providing innovative solutions to diverse clinical challenges (6–8). Thus, there is an urgent need for a comprehensive, systematic, and clear understanding of the current international development status of CDSS.

Research on CDSS began in the late 1950s, culminating in the development of the world’s first CDSS at Stanford University in the mid-1970s (9). With the rapid advancement of electronic medical record (EMR) systems and artificial intelligence (AI), CDSS has increasingly become a significant application of AI in the medical field (10). The integration of AI technologies, including machine learning, neural network algorithms, and decision trees, with traditional CDSS allows for the rapid transformation of complex patient information into concise, organized data, facilitating support for clinical decision-making (11).

Quantitative research utilizing knowledge graphs can objectively illustrate the hotspots and emerging development trends within a specific research domain. To effectively assess the research status of CDSS, this article employs CiteSpace visualization software to analyze relevant literature from the past decade on an international scale. It aims to summarize the current research landscape, identify key hotspots, and outline future development directions in this field. This comprehensive understanding of CDSS is intended to serve as a reference for ongoing research and the sustainable advancement of CDSS in the future.

## 2 Data and methods

### 2.1 Source of information

This study utilized the Web of Science (WOS) core database as its primary literature source. The search period spanned from January 1, 2014, to May 1, 2024. A topic search was conducted using the formula TS = clinical decision support system OR clinical decision support systems, with filters applied for language (English) and literature type (article). The search results were subsequently exported in plain text format. Following a manual review to exclude irrelevant literature and deduplication, a total of 2,668 articles were obtained.

### 2.2 Research methods

In this study, the relevant literature was exported to the Web of Science (WOS) in plain text format and subsequently imported into the bibliometric software CiteSpace (version 6.2.R4). The analysis period (Time Slice) for CiteSpace is set from 2014 to 2024, with a single time partition (year per slice) configured to 1 year. The analysis aims to visualize and generate a graph based on various factors, including country, author, institution, cited literature, keywords, and keyword clustering.

## 3 Results

### 3.1 Analysis of publication volume

**FIGURE 1:**
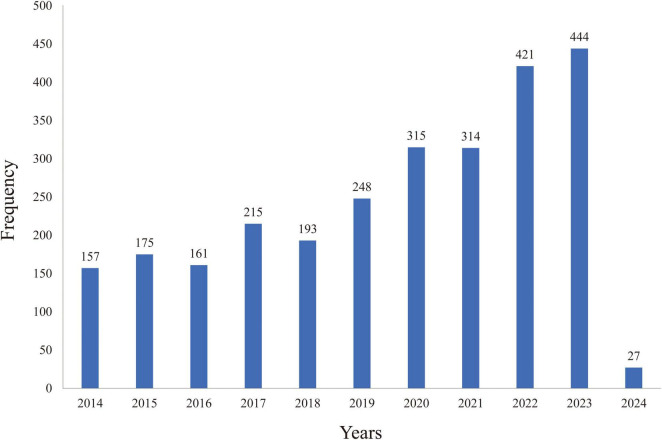
Annual publication volume.

According to literature statistics from 2014 to 2024, the number of research articles on clinical decision support systems has demonstrated a consistent upward trend, rising from 157 in 2014 to 444 in 2023—more than doubling in this period. Especially in 2023, there will be a peak in publication volume. This indicates that research on clinical decision support systems is garnering increasing attention from the academic community. The distribution of research publications on clinical decision support systems is illustrated in Figure 1.

### 3.2 Analysis of national cooperation

**FIGURE 2:**
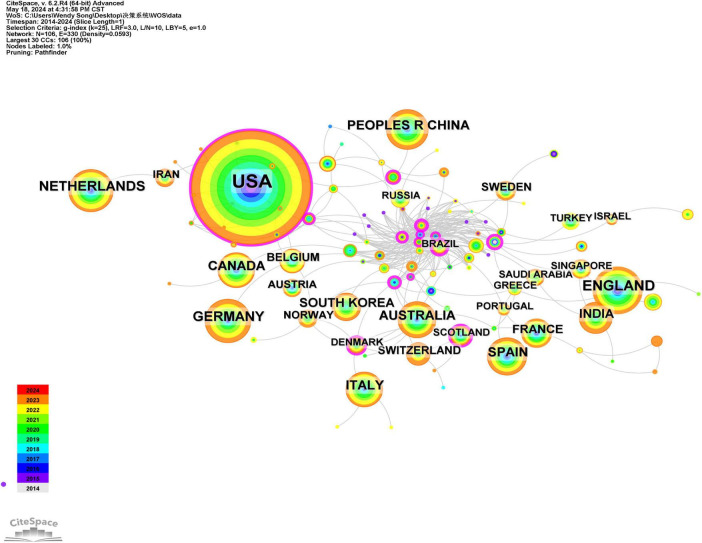
The national collaborative analysis atlas of clinical decision support systems in the WOS core set database from 2014 to 2024.

Using CiteSpace software for analysis with countries as nodes, the results indicate that a total of 105 countries have published relevant literature. The top three countries in terms of publication frequency are the United States (1,108 articles), the United Kingdom (223 articles), and Germany (185 articles), which together account for 39.15% of the total publication volume. The top 10 countries in terms of publication volume collectively represent 63.95% of the total output (Table 1). The cooperation graph comprises 106 nodes and 330 connections, reflecting strong collaborative relationships among countries (Figure 2).

**TABLE 1:** Top 10 countries with the highest number of publications on decision support systems in the WOS database from 2014 to 2024.

Sort	Count	No. of publications	Percentage (%)
1	U.S.A	1,108	28.62
2	England	223	5.76
3	Germany	185	4.78
4	Netherlands	181	4.67
5	China	142	3.67
6	Spain	137	3.54
7	Italy	130	3.36
8	Australia	130	3.36
9	Canada	130	3.36
10	India	110	2.84

### 3.3 Author collaboration analysis

**FIGURE 3:**
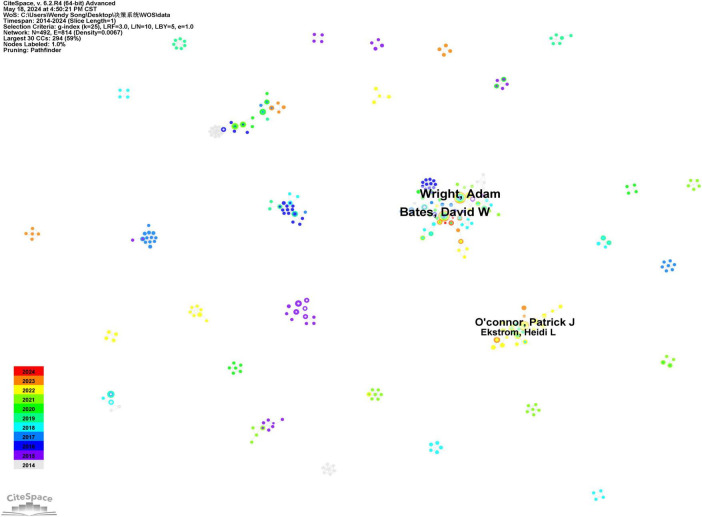
Author collaboration analysis graph of clinical decision support system in WOS core set database from 2014 to 2024.

Using CiteSpace software, 491 authors have published literature related to clinical decision support systems, with the authors represented as nodes. The top three authors in terms of publication volume are Professor Wright and Adam from the Department of Biomedical Information at Vanderbilt University in the United States, Professor Bates and David W from Brigham and Women’s Hospital in the United States, and Professor O’Connor and Patrick J from the School of Education at the University of Georgia. Professor Wright and Adam have published 30 articles, ranking first. Their research primarily focuses on analyzing failures in clinical decision support systems and clinical medication safety warning reminders (12). Professor Bates, David W has published 29 articles, mainly concentrating on clinical decision support system fault analysis, electronic health records, and adverse drug event alerts (13). Professor O’Connor and Patrick J have published 19 articles, primarily focusing on the application of clinical decision support systems in managing chronic diseases such as hypertension, cardiovascular risk, diabetes, chronic kidney disease, and cancer prevention (14). The collaboration graph indicates a close working relationship between Professors Wright and Adam and Professors Bates and David W (15) (Figure 3).

### 3.4 Analysis of institutional cooperation

**FIGURE 4:**
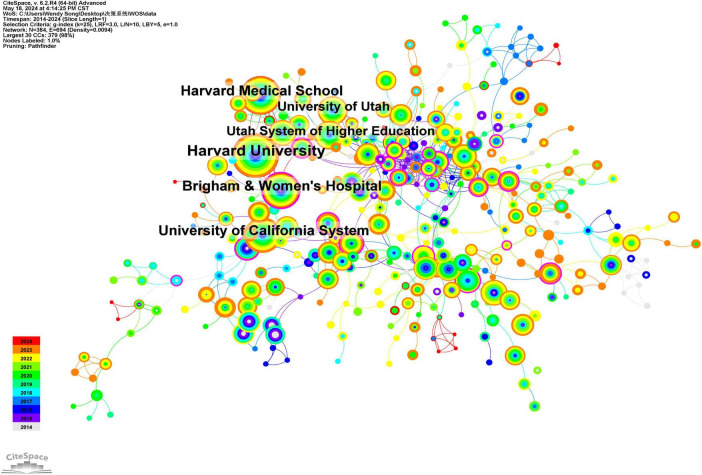
Clinical decision support system institutional collaboration analysis atlas in the WOS core set database from 2014 to 2024.

Using CiteSpace software, a total of 384 institutions published relevant literature, with these institutions represented as nodes. The top five institutions by publication volume are Harvard University, with 165 articles; Harvard Medical School, with 117 articles; the University of California System, with 110 articles; Brigham and Women’s Hospital, with 107 articles; and the Utah System of Higher Education, with 69 articles. These five institutions account for 12.79% of the total number of publications, while the top 10 institutions collectively account for 19.68% of the total publications, as illustrated in Table 2. Figure 4 depicts a collaboration graph that includes 384 nodes and 694 connections. The publishing institutions demonstrate close cooperation, particularly among the top five, which are all located in the same country, facilitating convenient collaboration.

**TABLE 2:** Top 10 institutions with the highest number of publications on decision support systems in the WOS database from 2014 to 2024.

Rank	Institution	No. of publications	Percentage (%)
1	Harvard University	165	3.71
2	Harvard Medical School	117	2.63
3	University of California	110	2.48
4	Brigham and Women’s Hospital	107	2.41
5	Utah Higher Education System	69	1.55
6	University of Utah	69	1.55
7	University of Pennsylvania	62	1.40
8	Vanderbilt University	60	1.35
9	University of London	58	1.31
10	Department of Veterans Affairs	57	1.28

### 3.5 Citation analysis of cited literature

**FIGURE 5:**
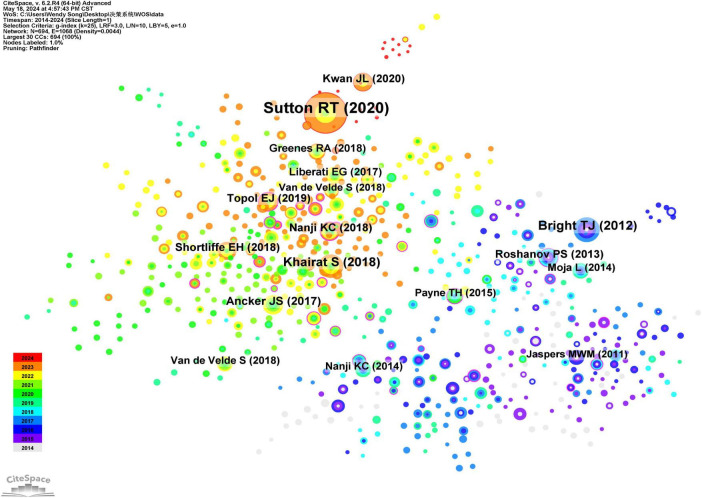
Collaborative analysis graph of highly cited coreliterature in the clinical decision support system of WOS core set database from 2014 to 2024.

Reference citation refers to one reference is cited by at least two other references, allowing for the prediction of research hotspots and trends within a specific timeframe in a given research field. Utilizing CiteSpace software to analyze the cited literature as nodes, a total of 694 nodes and 1,068 connections were identified (Table 3; Figure 5). Between 2014 and 2024, 694 research papers in this domain were cited as core references. The top five cited references are ranked as follows: the first is a review of clinical decision support systems, which provides an overview of their application in medicine, encompassing various types, effective use cases, common pitfalls, and potential hazards. Evidence-based recommendations are proposed to mitigate risks in the design, implementation, evaluation, and maintenance of CDSS. The second most frequently cited article is a systematic review that discusses the role of CDSS, indicating that both commercially available and locally developed systems can effectively enhance various healthcare process measures. The third most frequently cited analysis primarily examines the reasons behind physicians’ reluctance to utilize CDSS, proposing two major models for CDSS design: user acceptance and system adaptive design models, along with input-output participation models. The fourth most frequently cited study is a retrospective cohort analysis investigating the impact of workload, work complexity, and repeated alerts on alert fatigue in CDSS, utilizing electronic health record data from 112 primary care physicians in outpatient settings. The findings reveal that clinicians are increasingly less likely to accept alerts due to the high volume of alerts received, particularly repeated ones. The fifth most frequently cited literature explores the relationship between computerized clinical decision support systems and absolute improvements in nursing, employing a meta-analysis.

**TABLE 3:** The top 10 references with the highest citation frequency for decision support systems in the WOS database from 2014 to 2024.

Rank	Cited literature	Times cited	Percentage (%)
1	Sutton RT, 2020, NPJ Digit Med	186	4.70
2	Bright TJ, 2012, Ann Intern Med	69	1.75
3	Khairat S, 2018, JMIR Med Inf	61	1.54
4	Ancker JS, 2017, BMC Med Inform Decis	44	1.11
5	Kwan JL, 2020, BMJ-Brit Med J	39	0.99
6	Topol EJ, 2019, Nat Med	38	0.96
7	Nanji KC, 2018, J Am Med Inform Assn	37	0.94
8	Roshanov PS, 2013, BMJ-Brit Med J	37	0.94
9	Shortliffe EH, 2018, JAMA-J Am Med Assoc	37	0.94
10	Greenes RA, 2018, J Biomed Inform	33	0.83

### 3.6 Keyword colinear graph

**FIGURE 6:**
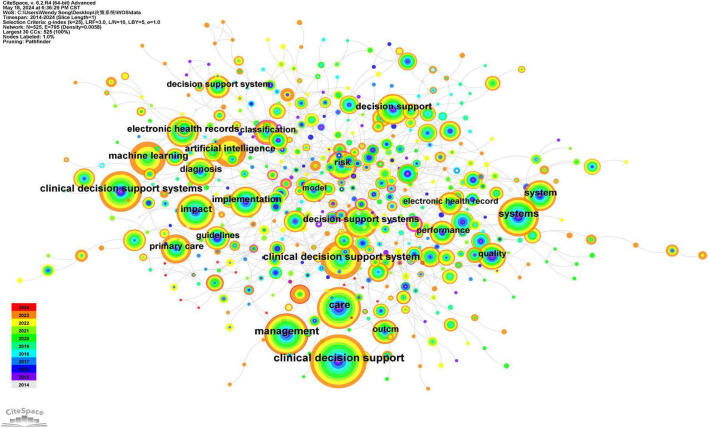
WOS core set database clinical decision support system keyword collinear network from 2014 to 2024.

Keywords are essential components of an article, reflecting the prevailing topics within the relevant field. Utilizing CiteSpace software for literature analysis, we identified 525 nodes and 795 connections (Figure 6). The top 20 keywords, based on the frequency of clinical decision support system-related literature published from 2014 to 2024, are summarized in Table 4. The keywords with high frequency of occurrence are categorized into three groups: ① Purpose of establishing CDSS—including clinical decision support, care, management, decision support, electronic health records, and primary healthcare; ② Functional Status and Design Optimization—encompassing systems, machine learning, decision support systems, and artificial intelligence; ③ Application situation—addressing impact, implementation, diagnosis, risk, and quality.

**TABLE 4:** The top 20 keywords in the frequency of decision support systems in the WOS database from 2014 to 2024.

Rank	Keywords	Frequency
1	Clinical decision support	483
2	Care	321
3	Management	301
4	Clinical decision support systems	282
5	Clinical decision support system	273
6	Systems	271
7	Impact	241
8	Machine learning	221
9	Decision support systems	191
10	Decision support	182
11	Artificial intelligence	177
12	Electronic health records	175
13	Implementation	175
14	System	167
15	Primary care	157
16	Diagnosis	156
17	Risk	141
18	Quality	130
19	Performance	122
20	Outcome	120

### 3.7 Keyword clustering analysis

**FIGURE 7:**
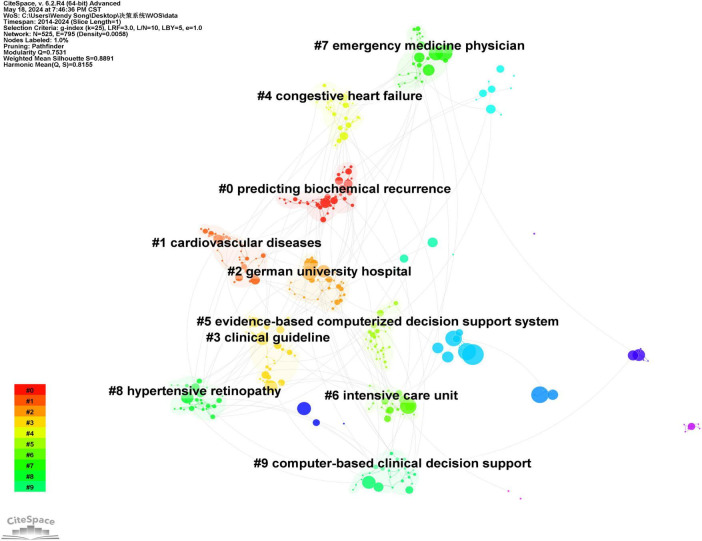
Keyword clustering map of clinical decision support system in WOS core set database from 2014 to 2024.

Based on the collinearity of keywords, a cluster analysis is conducted to group keywords into several clusters according to their thematic relevance. Each cluster represents a distinct focus within its respective research field, with larger clusters containing a greater number of keywords. The Q-value for keyword clustering in literature is 0.7531, and the S-value is 0.8891. This indicates that the clustering is reasonable. The top 10 keyword clusters (Figure 7) are as follows: #0 predicting biochemical recurrence, #1 cardiovascular diseases, #2 German university hospital, #3 clinical guidelines, #4 congestive heart failure, #5 evidence-based computerized decision support system, #6 intensive care unit, #7 emergency medicine physician, #8 hypertensive retinopathy, and #9 computer-based clinical decision support.

### 3.8 Timeline chart

**FIGURE 8:**
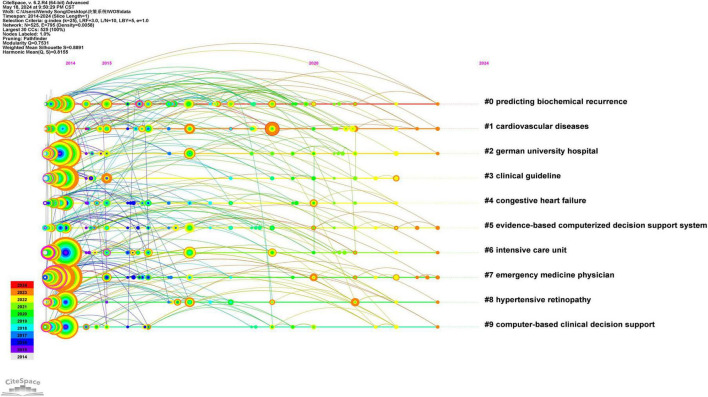
WOS core set database clinical decision support system keyword timeline from 2014 to 2024.

The timeline for publishing papers related to clinical decision support systems from 2014 to 2024 indicates that keywords such as “diagnosis,” “management,” and “alerts” were prominent in 2014. In contrast, keywords like “digital health,” “natural language processing,” and “implementation science” emerged only in 2020 (Figure 8).

## 4 Discussion

According to the graph, the publication of research papers related to CDSS has demonstrated a consistent upward trend from 2014 to 2024. Prior to 2017, the growth rate was relatively slow; however, following a brief decline in 2018, the rate of increase has accelerated since 2019. Since 2014, the number of publications has consistently surpassed 150, reflecting a sustained rise in the international research community’s interest in CDSS. Regarding the countries of publication, there is a notable prevalence of contributions from European and American nations, particularly the United States, the United Kingdom, and Germany. This trend may be attributed to the early advancement of computer technology and the initial implementation of electronic medical record systems in these countries, which have fostered a conducive environment for the integration and evolution of CDSS (16, 17). In terms of authors and institutions, four CDSS institutions have each published over 100 articles. These institutions are located in the same country and maintain close collaborative ties; however, the collaboration among authors appears to be relatively fragmented. Globally, particularly in low- and middle-income countries, there remains a pressing need to further develop high-quality, shared, and open large-scale disease databases. Additionally, the organic integration of CDSS with other physical systems should be reinforced to enhance its adaptability. Furthermore, multi-disciplinary collaboration should be promoted to fully harness the potential and advantages of CDSS in disease management.

The concept of CDSS was first proposed in the 1960s (18, 19). Since 2014, the development of algorithm models for integrating CDSS with hospital electronic medical records has significantly increased (20–23). In May 2022, the DECIDE-AI expert group officially released the Guidelines for Early Clinical Evaluation Reports of AI-based Clinical Decision Support Systems (DECIDE-AI), which provide a comprehensive list of operational items for the early clinical evaluation of the actual performance and safety of CDSS (24, 25).

The results of the evolution analysis of cited literature, including keyword collinearity, clustering, mutation, and time dimension, indicate that CDSS research currently encompasses various fields such as hypertension, cardiovascular risk, diabetes, chronic kidney disease, cancer, rare diseases, and drug safety. This research predominantly manifests in the form of electronic alerts, clinician advice packages, and patient data reports, which serve as resources for clinicians. A substantial body of literature has validated the intervention effects of CDSS from diverse perspectives, including model performance, clinical behavior improvement, and patient prognosis (2–5).

CDSS can be categorized into two primary types based on their system structure. The first category is knowledge-based CDSS, which comprises three main components: a knowledge base, an inference engine, and a human-machine communication interface. Due to its closed nature and the absence of deep learning capabilities, this type of CDSS necessitates manual completion of data collection, compilation, organization, and rule establishment. Consequently, this results in high maintenance costs and limited timeliness for information updates (26). The second category consists of non-knowledge-based CDSS, often implemented in the form of artificial intelligence. These systems utilize artificial neural networks equipped with machine learning capabilities, allowing them to summarize and clarify knowledge through human-computer interaction and continuous training. They leverage knowledge data to offer suggestions to users. By delivering accurate decision recommendations through their efficient learning capabilities, non-knowledge-based CDSS represents a significant trend for future development (27).

According to the evaluation of module value and average contour value in keyword clustering analysis, a module value Q > 0.3 indicates a significant clustering structure, while an average contour value S > 0.7 suggests a strong clustering correlation and convincing results (28). The keyword clustering analysis conducted in this study yielded a Q-value of 0.753 and an S-value of 0.889, which underscores the reliability of the clustering outcomes. These results are consistent with the co-occurrence and mutation patterns observed in the keywords. Notably, clinical guidelines, evidence-based computerized decision support systems (CDSS), and computer-based clinical decision support have emerged prominently within the clustering results. Both domestic and international studies have demonstrated that guideline-based CDSS plays a crucial role in enhancing the quality of medical and nursing services, thereby improving patient clinical outcomes. CDSS is expected to leverage computer technologies such as artificial intelligence and machine learning to analyze and process extensive medical data—including clinical data, physiological parameters, genetic information, and imaging data—thus improving the accuracy and effectiveness of data analysis and decision support (29, 30). Moreover, greater emphasis should be placed on the user experience of CDSS, aiming to make system design more user-friendly and practical, which would facilitate easier interaction for clinical workers and enhance doctors’ recognition and acceptance of CDSS (31–33). In the future, artificial intelligence and machine learning will become important technologies for connecting massive medical data with precise treatment plans in the process of building CDSS (34). By deeply mining and analyzing massive medical database resources, integrating and utilizing heterogeneous data from multiple sources, a comprehensive disease knowledge network will be formed, and precise medical advice can be provided based on personalized patient characteristics. With the rapid growth of medical data and the rapid development of artificial intelligence technology, CDSS based on knowledge graph will more efficiently assist medical personnel in clinical decision-making.

## 5 Conclusion

CDSS-based disease diagnosis, system management, alerts, biochemical relapse prediction, clinical guidelines, and evidence-based computerized decision support systems are current research hotspots. Artificial intelligence, natural language processing, and user-centered design are the future trends.

## 6 Limitations

This study has limitations, due to its exclusive focus on literature from the WOS core database, which may introduce selection bias. Future research should continue to monitor the current status and development trends in CDSS research, while also exploring the refinement and analysis of relevant content across various subcategories of CDSS research.

## Data Availability

The original contributions presented in this study are included in this article/supplementary material, further inquiries can be directed to the corresponding author/s.
